# Hyperglycemia Promotes the Epithelial-Mesenchymal Transition of Pancreatic Cancer via Hydrogen Peroxide

**DOI:** 10.1155/2016/5190314

**Published:** 2016-06-28

**Authors:** Wei Li, Lun Zhang, Xin Chen, Zhengdong Jiang, Liang Zong, Qingyong Ma

**Affiliations:** Department of Hepatobiliary Surgery, First Affiliated Hospital of Xi'an Jiaotong University, Xi'an 710061, China

## Abstract

Diabetes mellitus (DM) and pancreatic cancer are intimately related, as approximately 85% of patients diagnosed with pancreatic cancer have impaired glucose tolerance or even DM. Our previous studies have indicated that high glucose could promote the invasive and migratory abilities of pancreatic cancer cells. We therefore explored the underlying mechanism that hyperglycemia modulates the metastatic potential of pancreatic cancer. Our data showed that streptozotocin- (STZ-) treated diabetic nude mice exhibit larger tumor size than that of the euglycemic mice. The number of nude mice that develop liver metastasis or ascites is much more in the STZ-treated group than that in the euglycemic group. Hyperglycemic mice contain a higher plasma H_2_O_2_-level than that from euglycemic mice. The injection of polyethylene glycol-conjugated catalase (PEG-CAT), an H_2_O_2_ scavenger, may reverse hyperglycemia-induced tumor metastasis. In addition, hyperglycemia could also modulate the expression of epithelial-mesenchymal transition- (EMT-) related factors in pancreatic tumor tissues, as the E-cadherin level is decreased and the expression of mesenchymal markers N-cadherin and vimentin as well as transcription factor snail is strongly increased. The injection of PEG-CAT could also reverse hyperglycemia-induced EMT. These results suggest that the association between hyperglycemia and poor prognosis of pancreatic cancer can be attributed to the alterations of EMT through the production of hydrogen peroxide.

## 1. Introduction

As the fourth leading cause of cancer death worldwide, pancreatic cancer (PC) is an aggressive and intractable malignant disease due to the lack of early symptoms, poor prognosis, short survival, and resistance to therapy [1]. Approximately 75% of PC patients die within 1 year of diagnosis and only 5% or less survive for 5 years [2]. It has been projected that PC will become the leading cause of cancer-related deaths in the USA by 2050 [3]. In China, it has been estimated that 90,100 subjects will be newly diagnosed with PC and will account for 79,400 cancer-related death in 2015 [4]. Due to the fact that almost 80% of PC patients have locally deteriorated or metastatic disease, they are not appropriate for resection in the early stage of tumor development [5]. It is important to highlight cellular mechanisms of the etiological and risk factors to this disease which might lead us to find more effective therapeutic strategies.

Diabetes mellitus (DM), a major worldwide public health problem, is associated with certain site-specific cancers, including liver, biliary tract, pancreatic, and colorectal cancer [6–9]. In recent years, DM has been postulated to be both an independent risk factor and a consequence for PC and up to 80% of PC patients have pathologic glucose tolerance or DM test at diagnosis [10]. Our previous* in vitro* studies showed that high glucose (HG) can be regarded as an accelerator to increase cell proliferation through enhanced epidermal growth factor (EGF)/EGFR signaling [11]. We have also proven that hyperglycemia may worsen the prognosis of PC by enhancing their migratory and invasive ability through the production of hydrogen peroxide (H_2_O_2_) [12], which might be modulated by the expression of superoxide dismutase (SOD) through the activation of the ERK and p38 MAPK signaling pathways [13]. In addition, we also demonstrated that DM enhances perineural invasion in PC patients and aggravates a poor prognosis [14]. The inner mechanism between PC metastasis and DM should be deeply evaluated.

Distant metastasis, considered as the pivotal step in solid tumor progression, is responsible for approximately 90% of cancer-related deaths [15]. The pathogenesis of cancer metastasis is complex and not fully understood. Epithelial-mesenchymal transition (EMT), which is originally established during embryogenesis, has been intimately related with cancer metastasis by allowing a polarized epithelial cell to assume a mesenchymal cell phenotype and gaining enhanced migratory and invasive capacity [10]. A typical symbol of EMT includes a striking decline in the cell-cell adhesion molecule E-cadherin expression and gain of mesenchymal markers, such as vimentin and N-cadherin, culminating in cell morphology change as well as enhanced cell motility [16]. Recently, accumulating data and studies have started to indicate the relationship between hyperglycemia and EMT, especially on diabetic renal injury [17, 18] and peritoneal dialysis [19, 20]. Our recent studies proved that H_2_O_2_ production can promote EMT in PC, leading to increased motility and invasion via activation of ERK signaling pathway [21]. However, whether hyperglycemic condition could influence EMT in PC has not been elucidated.

In the current study, we investigated the production of H_2_O_2_ in both STZ-treated diabetic nude mice and euglycemic nude mice. We also tested the hypothesis that H_2_O_2_ mediates hyperglycemia-induced EMT and regulates the metastatic activity of PC. Our findings may provide new insight on the relationship between DM and PC and reveal a novel therapeutic strategy for PC patients who suffer from diabetes.

## 2. Materials and Methods

### 2.1. Cell Culture and Reagents

The human PC cell lines Panc-1, obtained from the American Type Culture Collection (Manassas, VA, USA), were cultured in Dulbecco's Modified Eagle's Medium (DMEM) containing 10% dialyzed heat-inactivated fetal bovine serum (FBS), 100 U/mL penicillin, and 100 *μ*g/mL streptomycin in a 95% air/5% CO_2_ humidified atmosphere at 37°C. DMEM and FBS were purchased from HyClone (Logan, UT, USA). Streptozotocin (STZ) and CAT derivative conjugated with polyethylene glycol (PEG-CAT) were acquired from Sigma Aldrich (St. Louis, MO, USA). The primary antibodies against E-cadherin, N-cadherin, vimentin, snail, and *β*-actin were purchased from Santa Cruz Biotechnology (Santa Cruz, CA, USA). The BCA assay kit was purchased from Pierce (Rockford, IL, USA). Other reagents were purchased from common commercial sources. All drug solutions were freshly prepared on the day of testing.

### 2.2. Diabetes Mouse Model

BALB/c athymic nude mice (male, 5 weeks old) were purchased from Shanghai Experimental Animal Center (Chinese Academy of Sciences, China). Animal care and experiments were carried out in accordance with guidelines of the Xi'an Jiaotong University. BALB/c athymic nude mice were grouped into euglycemia, hyperglycemia, euglycemia + PEG-CAT, and hyperglycemia + PEG-CAT groups (*n* = 6), of which hyperglycemia mice received an intraperitoneal injection of STZ dissolved in sodium citrate buffer (pH 4.5) at a dose of 175 mg/kg body weight. Blood glucose levels were determined with an ACCU-CHEK Active meter (Hoffmann-La Roche, Basel, Switzerland).

### 2.3. Orthotopic Tumor Model

At 2 weeks after STZ injection, Panc-1 cells (1 × 10^8^) were injected in a total volume of 50 *μ*L PBS into the body of the pancreas. 3 days later, a dose of 1000 units/d PEG-CAT was intraperitoneally injected into nude mice (euglycemia + PEG-CAT and hyperglycemia + PEG-CAT groups). The mice were sacrificed after 8 weeks and the tumors, liver, spleen, pancreas, and blood were collected and analyzed. Tumor volumes were determined by using the formula width^2^  ×  length × 0.5.

### 2.4. Hydrogen Peroxide Assay

The level of mice plasma H_2_O_2_ was measured using a H_2_O_2_ assay kit (Beyotime Institute of Biotechnology, Jiangsu, China) according to the manufacturer's instructions. In this kit, the ferrous ions Fe^2+^ were oxidized to ferric ions Fe^3+^ by H_2_O_2_. The ferric ions further formed a complex with the indicator dye xylenol orange and produced a visible purple-colored complex that could then be measured using a microplate reader at a wavelength of 560–590 nm (Bio-Rad, CA, USA) [22].

### 2.5. Real-Time Quantitative PCR (qRT-PCR)

Total RNA was extracted from the orthotopic tumor of pancreas using the Fastgen200 RNA isolation system (Fastgen, Shanghai, China) according to the manufacturer's protocol. Total RNA was reverse-transcribed into cDNA using a PrimeScript RT reagent Kit (TaKaRa, Dalian, China). The primer sequences were as follows:

 E-Cadherin-F: 5′-ATT CTG ATT CTG CTG CTC TTG-3′.
 E-Cadherin-R: 5′-AGT CCT GGT CCT CTT CTC C-3′.
 N-Cadherin-F: 5′-ATG GTG TAT GCC GTG AGA AG-3′.
 N-Cadherin-R: 5′-TGT GCT TAC TGA ATT GTC TTG G-3′.
 Vimentin-F: 5′-AAT GAC CGC TTC GCC AAC-3′.
 Vimentin-R: 5′-CCG CAT CTC CTCCTC GTA G-3′.
 Snail-F: 5′-CTT CTC CTC TAC TTC AGT CTC TTC-3′.
 Snail-R: 5′-CGT GTG GCT TCG GAT GTG-3′.
 
*β*-actin-F: 5′-GAC TTA GTT GCG TTA CAC CCT TTC T-3′.
 
*β*-actin-R: 5′-GAA CGG TGA AGG TGA CAG CAG T-3′.

After each qRT-PCR, a dissociation curve analysis was conducted. Relative gene expression was calculated using the 2^−ΔΔCt^ method reported previously [23]. Each measurement was carried out in triplicate.

### 2.6. Western Blotting

After being electrophoretically resolved on a denaturing SDS-polyacrylamide gel, proteins were electrotransferred onto polyvinylidene difluoride (PVDF) membranes. The membranes were initially blocked with 5% nonfat dry milk in Tris-buffered saline (TBS) for 2 h and then probed with antibodies against E-cadherin, N-cadherin, vimentin, snail, or *β*-actin as loading control. After coincubation with the primary antibodies at 4°C overnight, membranes were incubated with the secondary antibody for 2 h at room temperature. The results were visualized using the ECL Western blotting substrate and photographed by GeneBox (SynGene).

### 2.7. Immunohistochemistry

Formalin fixed and paraffin embedded orthotopic pancreatic tumor tissue samples were used for the immunohistochemistry test. In brief, the tissue sections were incubated with primary antibodies (anti-E-cadherin, anti-N-cadherin, anti-vimentin, and anti-snail, 1 : 50) overnight at 4°C and incubated with the appropriate biotinylated secondary antibody for 30 min at room temperature. After rinsing, the results were visualized using diaminobenzidine (DAB) and the slides were counterstained with hematoxylin. The densitometry analysis of immunohistochemical staining was performed using the Image-Pro Plus 6.0 software.

### 2.8. Statistical Analysis

Statistical analysis was performed using SPSS software (version 17.0, SPSS Inc., Chicago, USA). Data were presented as the means ± SEM of three replicate assays. Differences between the groups were assessed by analysis of Chi-square test and analysis of variance (ANOVA). Statistical significance was set at *P* < 0.05. All experiments were repeated independently at least three times.

## 3. Results

### 3.1. STZ Treatment Increases the Fasting Blood Glucose Levels and Decreases the Body Weight of the Nude Mice

**Figure 1:**
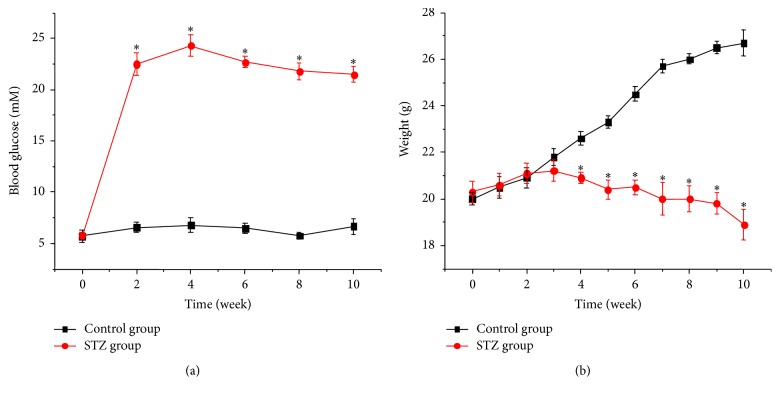
Effect of STZ on blood glucose and weight in nude mice. (a) Blood glucose in STZ-treated mice (*n* = 12). (b) Body weight in STZ-treated mice (*n* = 12). ∗ refers to *P* < 0.05 as compared with control group.

To determine the efficacy of different drugs against transplantation-established human tumor xenografts in the athymic nude mice, we used an orthotopic tumor model. STZ is a chemical which is commonly used to induce experimental diabetes in animals [24]. The characteristics of the STZ-treated nude mice used in this study were summarized in Figure 1. The fasting blood glucose levels were significantly increased from 2 weeks to 4 weeks and keep a high level till 10 weeks after STZ injection (Figure 1(a)). The body weight of the nude mice were reduced at 4 weeks after STZ injection (Figure 1(b)).

### 3.2. Hyperglycemia Enhances the Production of H_2_O_2_ in Mice Plasma

**Figure 2:**
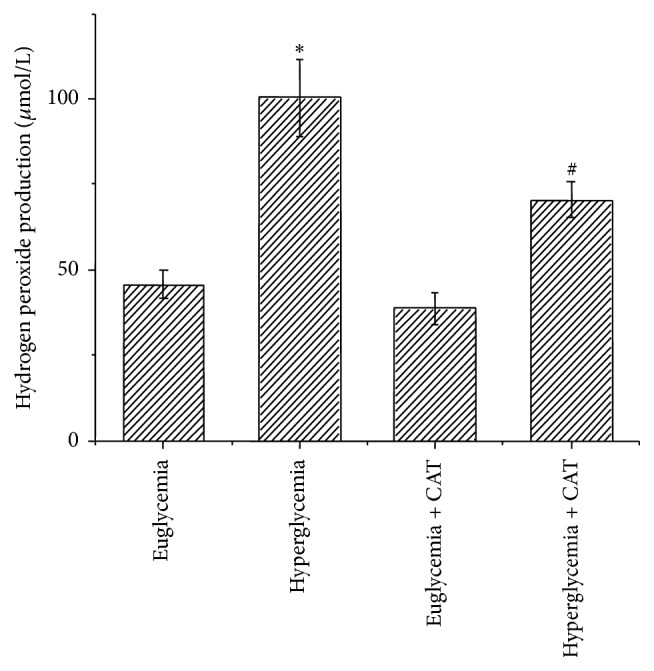
Effect of hyperglycemia on H_2_O_2_ production in mice plasma. The levels of H_2_O_2_ in mice plasma were tested before the mice were sacrificed. ^*∗*^ *P* < 0.05 as compared with euglycemia group; ^#^ *P* < 0.05 as compared with hyperglycemia group.

Hyperglycemic condition has been shown to induce the overproduction of reactive oxygen species (ROS), which consists of a number of chemically reactive molecules derived from oxygen, including H_2_O_2_ [25]. Our previous* in vitro* study also proved that H_2_O_2_ is able to mediate high glucose-induced invasive activity via ERK and p38 MAPK in human PC cells [13]. In this study, we tested the level of H_2_O_2_ in mice plasma before the mice were sacrificed. As shown in Figure 2, hyperglycemic mice contained a higher plasma H_2_O_2_ level than that from euglycemic mice. PEG-CAT could significantly reduce the blood H_2_O_2_ level of STZ-injected mice.

### 3.3. Hyperglycemia Induces Pancreatic Tumor Growth in Nude Mice

**Figure 3:**
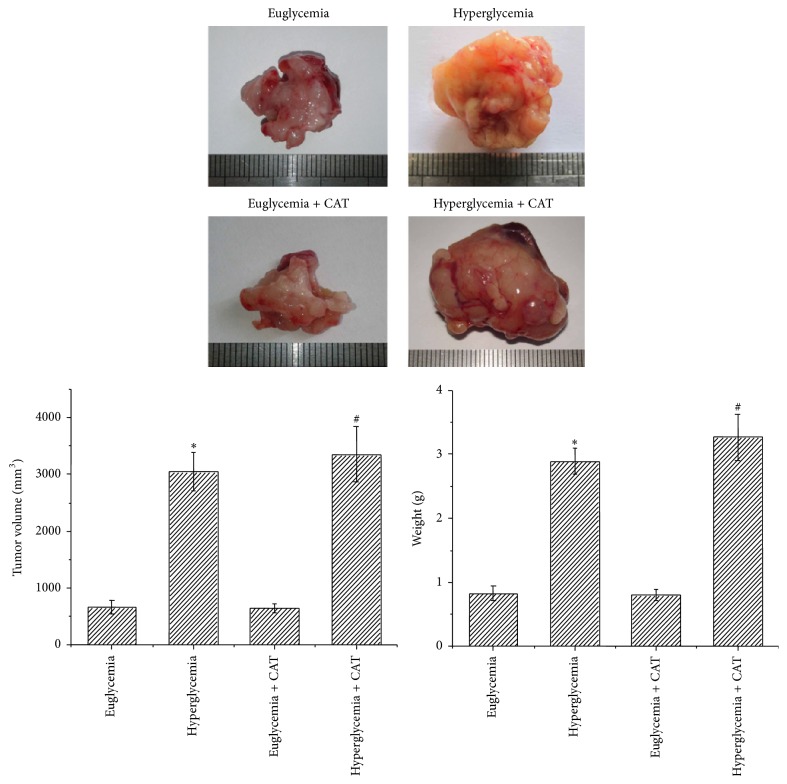
Effect of hyperglycemia on tumor growth in nude mice. Macroscopic appearance of solid tumors as well as tumor volumes and weights were tested after mice were sacrificed. ^*∗*^ *P* < 0.05 as compared with euglycemia group; ^#^ *P* < 0.05 as compared with euglycemia + CAT group.

Our previous study has proven that high glucose (25, 50 mM) could significantly increase the proliferation of PC cells compared with low glucose (5.5 mM) via the induction of EGF expression and transactivation of EGFR. The stimulating effect on cell proliferation in PC may be through the effect of accelerating cell cycle progression [11]. Here we found that the tumor volume and weight were increased in hyperglycemic mice than those in euglycemic mice. To evaluate whether the promotion of tumor growth is associated with the production of H_2_O_2_, mice were treated with PEG-CAT. As shown in Figure 3, the tumor volume and weight of hyperglycemic mice did not change after PEG-CAT injection.

### 3.4. Hyperglycemia Promotes Ascites Production and Liver Metastasis via H_2_O_2_ Production in PC

**Figure 4:**
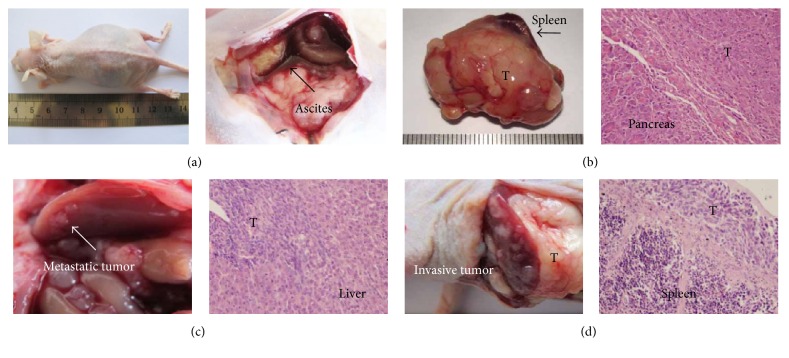
The metastasis of pancreatic tumor in nude mice. Metastatic Panc-1 tumors were analyzed by hematoxylin-eosin staining. (a) Ascites generation; (b) pancreatic tumor; (c) liver metastatic tumor; (d) spleen metastatic tumor. Original magnification ×400.

To determine whether hyperglycemic condition and H_2_O_2_ were involved in PC metastasis, equal numbers (1 × 10^8^) of Panc-1 cells were injected into the body of mice pancreas (both euglycemic mice and hyperglycemic mice). 3 days later, a dose of 1000 units/d PEG-CAT was intraperitoneally injected into nude mice. Eight weeks after injection of the cells, mice were sacrificed and the metastatic tumors were recorded (Figure 4). As described in Table 1, only one out of six euglycemic animals generated ascites, whereas five out of six hyperglycemic mice generated ascites. Two mice in the hyperglycemia + PEG-CAT group produced ascites. In addition, none of the euglycemic mice developed visible liver metastasis, whereas four out of six hyperglycemic mice developed liver metastasis. After injected PEG-CAT, only one hyperglycemic mouse developed liver metastasis. Taken together, these results show that the number of mice that develop liver metastasis or ascites is much more in the STZ-treated group than that in the euglycemic group. PEG-CAT injection might reverse these effects. The hyperglycemia-induced level of H_2_O_2_ might be involved in the acceleration of tumor metastasis.

### 3.5. Hyperglycemia Induces EMT via H_2_O_2_ Production in PC

**Figure 5:**
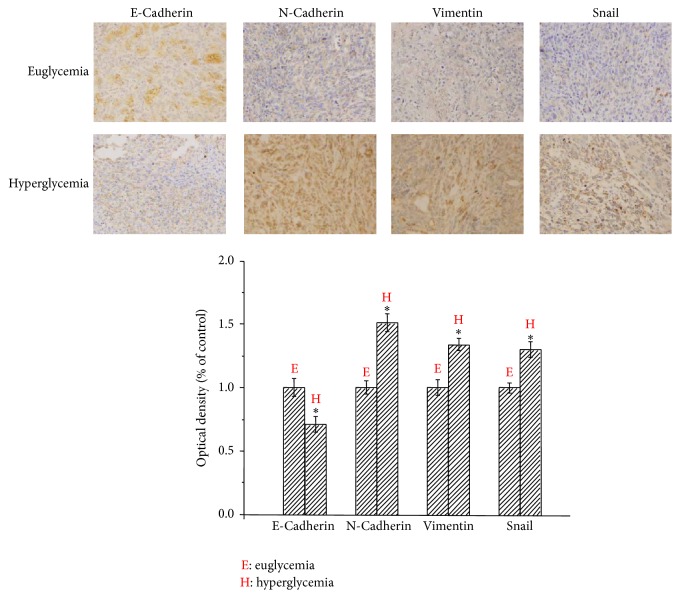
Effect of hyperglycemia on the expression of E-cadherin, N-cadherin, vimentin, and snail in nude mice. Immunohistochemistry was performed to compare the expression of E-cadherin, N-cadherin, vimentin, and snail in the orthotopic nude mice between euglycemia group and hyperglycemia group. ^*∗*^ *P* < 0.05 as compared with the euglycemia group. Original magnification ×400.

To further confirm whether hyperglycemia could induce EMT in PC, we examined the expression of EMT markers in the tumor tissue using immunohistochemical staining. As illustrated in Figure 5, the expression of E-cadherin was located in cell membrane, whereas N-cadherin, vimentin, and snail were mainly localized in cytoplasm. The E-cadherin staining of tumor cells was stronger in the euglycemia group than that in the hyperglycemia group, indicating that hyperglycemia was able to decrease the expression of E-cadherin. The percentage of cancer area with positive E-cadherin staining cancer cells was higher in the euglycemia group than that in the hyperglycemia group. In contrast, the N-cadherin, vimentin, and snail staining in the cytoplasm of the cancer cells was significantly stronger in the hyperglycemia group than that in the euglycemia group.

**Figure 6:**
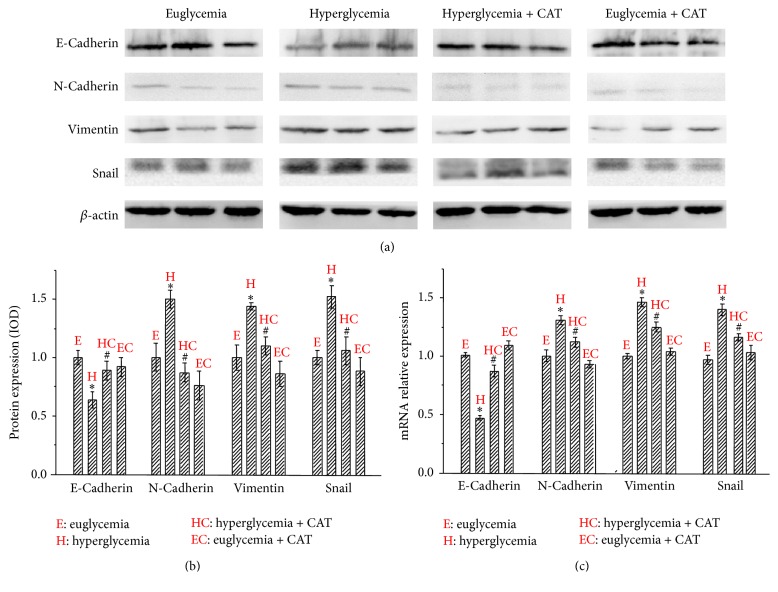
Effect of the hyperglycemia/H_2_O_2_ axis on EMT in nude mice. (a) The protein levels of EMT-related factors in pancreatic tumor tissues with different serum glucose levels were analyzed using Western blotting. (b) The statistical diagram of Western blotting analysis. (c) The mRNA levels of EMT-related markers in pancreatic tumor tissues with different serum glucose levels were analyzed using qRT-PCR. ^*∗*^ *P* < 0.05 as compared with the euglycemia group. ^#^ *P* < 0.05 as compared with hyperglycemia group.

To determine whether hyperglycemia-induced EMT was H_2_O_2_-related, we analyzed the expression of E-cadherin, N-cadherin, vimentin, and snail using Western blotting analysis. As shown in Figures 6(a) and 6(b), the protein level of E-cadherin in hyperglycemia group was lower than that in the euglycemia group. The expression of mesenchymal markers N-cadherin and vimentin as well as transcription factor snail was stronger in hyperglycemia group. PEG-CAT injection could reverse these hyperglycemia-induced effects. We next evaluate the effects of hyperglycemia and PEG-CAT on the expression of E-cadherin, N-cadherin, vimentin, and snail at mRNA level by qRT-PCR. As shown in Figure 6(c), PEG-CAT counterbalanced hyperglycemia-induced EMT-related factors at the mRNA level and the trend was consistent with the protein results. Taken together, our results demonstrate that hyperglycemia could induce EMT progression and facilitate tumor metastasis via the production of H_2_O_2_ in PC.

## 4. Discussion

As one of the most lethal malignant diseases, PC is characterized by early invasion and metastasis, which partially account for a compromised therapeutic effect and poor outcome [26]. In recent years, although the largest improvements in survival have been made for a number of cancers, PC still shows the least improvement [1]. Therefore, the exploration of risk factors and metastatic mechanisms might lead us to find more effective therapeutic strategies for PC.

DM, a common metabolic disorder characterized by hyperglycemia, has been postulated to be both an independent risk factor and a consequence for PC in recent years [27]. A meta-analysis of 6 case-control studies and 3 cohort studies showed that a 2-fold higher risk of PC was observed in type-1 DM patients compared with individuals without DM [28]. Another meta-analysis from three large case-control studies revealed a 1.8-fold increase in risk of pancreatic cancer associated with type-2 DM [11]. In addition, a recent meta-analysis of 3 case-control studies and 10 cohort studies showed that using of metformin appeared to be associated with a reduced risk of pancreatic cancer in patients with type-2 DM [29]. In Chinese Han people, a moderate increased risk of pancreatic cancer was discovered among cases with long-standing diabetes, with an AOR of 2.11 (1.51–2.94), while in the cases with new-onset DM (i.e., less than 24 months in duration), the AOR is 4.43 (3.44–5.72) compared to those without DM [30]. We have proven that glucose concentrations could alter the expression of glial cell line-derived neurotrophic factor and its tyrosine kinase receptor RET in a concentration-dependent manner, correspondingly with the alterations of cell proliferation [31]. Our previous study has also shown that high glucose may worsen the prognosis of pancreatic cancer by enhancing their migratory and invasive ability through SOD-induced H_2_O_2_ production via the activation of the ERK and p38 MAPK signaling pathways [12, 13]. In addition, the invasive ability of both the BxPC-3 and Panc-1 cells was strongly enhanced in the DM renal capsule xenograft model and this increase could be suppressed by PEG-CAT treatment [13]. In the current study, we showed that DM was able to promote liver metastasis or ascites production in the orthotopic tumor model.

EMT, a pivotal step in tumor metastasis, contains three essential processes: first, alterations of cell-cell and cell-extracellular matrix (ECM) interactions occur releasing the epithelial cells from the surrounding tissue. Then the cytoskeleton is reorganized so that the cells can gain the ability to move through ECM. After that, a new transcriptional program is induced to acquire morphological and functional characteristics of mesenchymal-like cells and gaining enhanced migratory and invasive capacity [10]. Our previous study has proven that SOD-induced H_2_O_2_ production can promote EMT in pancreatic cancer cells, leading to increased motility and invasion via activation of ERK signaling pathway [21]. The relationship between hyperglycemia and EMT has been revealed especially on diabetic renal injury and peritoneal dialysis. EMT contributes to the accumulation of matrix proteins in kidneys, in which renal tubular epithelial cells play an important role in progressive renal fibrosis. Kang et al. [32] revealed that high glucose could induce renal EMT through increasing expression of the mesenchymal markers vimentin, *α*-smooth muscle actin, and fibroblast-specific protein-1 in human renal proximal tubular epithelial cells and diabetic mice. A recent research also showed that HG could induce EMT in human lung adenocarcinoma epithelial A549 cells, as demonstrated by the secretion of TGF-*β*, cell morphology changes, the emergence of mesenchymal markers, and increased cellular motility [33]. In the current study, we showed that a single injection of STZ could lead to significant increase in fasting blood glucose in nude mouse. Hyperglycemic condition could promote tumor metastasis to liver and ascites production which might be attributed to the occurrence of EMT.

ROS generated by the mitochondrial respiratory chain consists of a number of chemically reactive molecules derived from oxygen, such as superoxide anion and H_2_O_2_. Accumulating evidence indicates that the intracellular redox state plays an important role in cellular signaling transduction and regulates multiple events, including tumor metastasis [34]. On one hand, an excessive amount of ROS production can kill cancer cells, whereas moderate concentrations of ROS can stimulate tumor progression by promoting cell proliferation, survival, invasion, and metastasis [35]. Our previous study has summarized that hyperglycemia is able to promote the invasive and migratory activity of BxPC-3 and Panc-1 cells via ROS production [12]. In order to confirm whether hyperglycemia-induced EMT is regulated by H_2_O_2_, we treated hyperglycemic mice with PEG-CAT that could eliminate H_2_O_2_. Our results confirm that hyperglycemia-induced H_2_O_2_ influences the metastasis ability via EMT in the pancreatic cancer. Recently, Ikemura et al. [36] proved that there were greater and more numerous tumor metastatic colonies in the lung and liver of the STZ-treated mice, and injections of PEG-CAT were effective in inhibiting tumor metastasis which was consistent with our results.

## 5. Conclusion

In conclusion, our results indicate that hyperglycemia is correlated with tumor size, liver metastasis, or ascites formation of pancreatic cancer. The hyperglycemia-induced enhanced metastasis ability might be attributed to the occurrence of EMT via the production of H_2_O_2_. Our findings may provide new insight on the relationship between DM and PC. Managing hyperglycemia/H_2_O_2_ axis might be a novel strategy for the treatment of this severe malignancy. Our findings warrant further investigation of this hypothesis.

## Figures and Tables

**Table 1:** The number of mice that develop liver metastasis, spleen metastasis, or ascites.

Groups (number)	Ascites	Liver metastasis	Spleen metastasis
Euglycemia (6)	1	0	1
Hyperglycemia (6)	5^*∗*^	4^*∗*^	2
Euglycemia + PEG-CAT (6)	0	0	0
Hyperglycemia + PEG-CAT (6)	2	1	0

^*∗*^ *P* < 0.05 as compared with euglycemia group.
